# Patient-specific dosimetry adapted to variable number of SPECT/CT time-points per cycle for $^{177}$Lu-DOTATATE therapy

**DOI:** 10.1186/s40658-022-00462-2

**Published:** 2022-05-16

**Authors:** Laure Vergnaud, Anne-Laure Giraudet, Aurélie Moreau, Julien Salvadori, Alessio Imperiale, Thomas Baudier, Jean-Noël Badel, David Sarrut

**Affiliations:** 1 CREATIS, CNRS UMR 5220, INSERM U 1044, Université de Lyon, INSA-Lyon, Université Lyon 1, Lyon, France; 2 Centre de lutte contre le cancer Léon Bérard, Lyon, France; 3 ICANS - Institut de cancérologie Strasbourg Europe, Strasbourg, France

**Keywords:** $^{177}$Lu-DOTATATE, Internal radiotherapy, Dosimetry, SPECT/CT, Single acquisition, Patient-specific, Monte Carlo simulation

## Abstract

**Background:**

The number of SPECT/CT time-points is important for accurate patient dose estimation in peptide receptor radionuclide therapy. However, it may be limited by the patient’s health and logistical reasons. Here,  an image-based dosimetric workflow adapted to the number of SPECT/CT acquisitions available throughout the treatment cycles was proposed, taking into account patient-specific pharmacokinetics and usable in clinic for all organs at risk.

**Methods:**

Thirteen patients with neuroendocrine tumors were treated with four injections of 7.4 GBq of $^{177}$Lu-DOTATATE. Three SPECT/CT images were acquired during the first cycle (1H, 24H and 96H or 144H post-injection) and a single acquisition (24H) for following cycles. Absorbed doses were estimated for kidneys (LK and RK), liver (L), spleen (S), and three surrogates of bone marrow (L2 to L4, L1 to L5 and T9 to L5) that were compared. 3D dose rate distributions were computed with Monte Carlo simulations. Voxel dose rates were averaged at the organ level. The obtained Time Dose-Rate Curves (TDRC) were fitted with a tri-exponential model and time-integrated. This method modeled patient-specific uptake and clearance phases observed at cycle 1. Obtained fitting parameters were reused for the following cycles, scaled to the measure organ dose rate at 24H. An alternative methodology was proposed when some acquisitions were missing based on population average TDRC (named STP-Inter). Seven other patients with three SPECT/CT acquisitions at cycles 1 and 4 were included to estimate the uncertainty of the proposed methods.

**Results:**

Absorbed doses (in Gy) per cycle available were: 3.1 ± 1.1 (LK), 3.4 ± 1.5 (RK), 4.5 ± 2.8 (L), 4.6 ± 1.8 (S), 0.3 ± 0.2 (bone marrow). There was a significant difference between bone marrow surrogates (L2 to L4 and L1 to L5, Wilcoxon’s test: *p* value < 0.05), and while depicting very doses, all three surrogates were significantly different than dose in background (*p* value < 0.01). At cycle 1, if the acquisition at 24H is missing and approximated, medians of percentages of dose difference (PDD) compared to the initial tri-exponential function were inferior to 3.3% for all organs. For cycles with one acquisition, the median errors were smaller with a late time-point. For STP-Inter, medians of PDD were inferior to 7.7% for all volumes, but it was shown to depend on the homogeneity of TDRC.

**Conclusion:**

The proposed workflow allows the estimation of organ doses, including bone marrow, from a variable number of time-points acquisitions for patients treated with $^{177}$Lu-DOTATATE.

## Introduction

Peptide receptor radionuclide therapy is a cancer treatment option [1] for patients suffering from unresectable neuroendocrine tumors that express somatostatin receptors [2]. The treatment consists to intravenously inject somatostatin analogue radiolabelled with $^{177}$Lu ($^{177}$Lu-DOTATATE). The treatment efficacy was demonstrated in the NETTER study [1], and its use was approved by the FDA in 2018 [3]. The standardized treatment is composed of four cycles of 7.4 GBq injections, about 8 weeks apart. However, it has been shown that the absorbed dose distribution is variable between patients [4], and the biodistribution of the radiopharmaceutical evolves over time after injection due to physiological mechanisms like the number of receptors or the blood flow in an organ [5]. Therefore, image-based personalized dosimetry can help to guide treatment and assess dose–response relationship [6–10].

Ideally, image-based absorbed doses estimation uses SPECT/CT acquired at several time-points after the injection to build Time Activity Curves (TAC) in organs of interest [11, 12]. However, in clinical practice, acquiring multiple time-points can be difficult due to patient health, technical or logistical reasons. Indeed, the standard procedure at our institution is to acquire three time-points for the first cycle (1H, 24H, 96H or 144H) and one single for the other cycles (24H).

In the literature, several articles propose dosimetric workflows requiring only a few acquisitions after each cycle. On the one hand, some works try to reduce the number of acquisitions by selecting the appropriate time-points and/or the type of acquisition (planar and/or SPECT/CT) so that the error from the original workflow is as small as possible [13, 14]. Others proposed workflows reduced to a single SPECT/CT acquisition from the second cycle [15–20], based on an approximation of the time-integrated activity or a priori information: use of pharmacokinetics obtained in a previous cycle [21] or from other patients [22]. In most published studies, the TAC is modelled with mono-exponential function that considers only one global decrease phase and not the three physiological phases: one uptake phase and two clearance phases (rapid- and long-term). Recently, Jackson et al. [22] developed a method based on a tri-exponential model to estimate the absorbed doses from a single activity time-point by using pre-calculated factors. These factors were obtained from selected time-activity curves of other patients and were time-dependent but not patient-specific. The method was validated for $^{177}$Lu-PSMA-617 therapy for different organs (kidneys, liver, spleen) and tumors but not for bone marrow. Moreover, in many articles, absorbed doses are computed using the MIRD formalism [23] based on pre-calculated S-values on digital phantoms [24]. However, cross-doses impact vary from patient to patient, and it is not easy to get S-values for tumors [25]. Overall, the proposed workflows are not fully patient-specific as they are based on pre-calculated S-values, the patient-specific pharmacokinetics are not respected or simplified by a mono-exponential function. Moreover, they are not validated for all organs at risk (OAR), and alternative methods were not necessarily proposed if acquisitions were missing from their standard procedures as it could be the case in clinical practice.

In this work, we propose an image-based patient-specific dosimetric workflow with a reduced number of acquisitions in $^{177}$Lu-DOTATATE therapy for neuroendocrine tumors applicable in clinic. Alternative methods were implemented and assessed in order to use the workflow even if some acquisitions are missing. The proposed method is based on Monte Carlo simulations to compute patient-specific dose rates. Time Dose-Rate Curves are fitted with tri-exponential functions and take into account patient-specific pharmacokinetics computed at one cycle with several time-points to estimate time-integrated absorbed dose for the other cycles with a single time-point. Also, several ways to evaluate dose in bone marrow, one of the two main OAR with kidneys [26], are proposed and compared.

## Material and methods

### Patients

This study considered thirteen patients, 8 women and 5 men, with neuroendocrine tumors (NET) treated by peptide receptor radionuclide therapy with $^{177}$Lu-DOTATATE (LUTATHERA$\circledR$), between November 2018 and March 2021 at the Léon Berard Center (Lyon, France). They were treated with four cycles of 6832 ± 158 MBq injections with about 8 weeks between two cycles, except for patient 1 with 28 weeks between cycles 3 and 4 and patient 10 with 24 weeks between cycles 2 and 3 and an injected activity equal to 3428 MBq for cycle 3. This cohort was named the *patient cohort*. Seven additional patients (2 women and 5 men) treated at the ICANS (Strasbourg, France) were considered. They received 7222 ± 113 MBq at cycles 1 and 4 between July 2020 and September 2021. In the following, this cohort will be named the *validation cohort*. Dosimetry was performed only for the patient cohort, whereas the validation of the proposed dosimetric methods was carried out with the patient and validation cohorts.

### Image acquisition

For the first cycle of the patient cohort, three tomographic SPECT/CT acquisitions were planned at 1H, 24H and 96H or 144H post-injection. The last time-point, 96H or 144H, varies according to the weekend constraints. For the other cycles, only one acquisition at 24H was planned. However, some acquisitions cannot be performed for clinical or technical reasons or due to the COVID pandemic. Table 1 summarizes the available images. A total of 63 images were considered. For patient P10, additional images were acquired at cycles 3 and 4.

**Table 1:** SPECT/CT acquisition time-points for all patients of the patient cohort, all cycles. NA: planned but not available, X: available

		P1	P2	P3	P4	P5	P6	P7	P8	P9	P10	P11	P12	P13
Cycle 1	1H	X	X	X	X	X	X	NA	X	X	X	NA	X	NA
	24H	X	X	X	X	X	X	X	X	NA	NA	X	X	X
	96H or	X	X	X	X	NA	X	NA	X	X	NA	X	NA	NA
	144H													
Cycle 2	24H	X	X	X	X	NA	NA	X	X	X	X	X	NA	X
Cycle 3	1H										X			
	24H	X	X	NA		NA	NA	X	X	X	X	X	X	X
	96H										X			
Cycle 4	1H								X	X	X			
	24H	X	X	NA		X	X	X			X	X	X	X
	96H										X			

For the validation cohort, three SPECT/CT acquisitions were performed and are available at 6H, at 24H and at 7 days after cycles 1 and 4.

### Quantitative tomographic SPECT/CT acquisitions

Image acquisitions were performed with a GE Discovery NM CT 670 for the patient cohort (GE Discovery NM CT 870 DR for the validation cohort). The imaging system is composed of two detectors at 180$^\circ$ equipped with MEGP (medium-energy general purpose) collimators and $3/8''$ thick crystal ($5/8''$ for the validation cohort). Each detector acquired 60 projections of 15 s per projection in step and shoot mode (30 projections of 40s for the validation cohort except for this acquisition at 7 days where the time of acquisition was doubled). The matrix had 128$\times$128 pixels of 4.4 mm. The photopeak energy window 208 keV was used with an energy window width of $15\%$ , and a 17.6% scatter correction window centered on 176.8 keV was applied ($20\%$ and $10\%$ for the validation cohort). Images were reconstructed in Xeleris 3.0 software with OSEM 3D algorithm (8 iterations and 8 subsets) with PSF correction (“resolution recovery”). Attenuation was corrected with the CT images. Scatter correction was performed with the dual-energy window method.

Pixel’s values in the reconstructed images, initially expressed in number of detected counts, were converted to quantitative activity in MBq/mL by using an experimentally determined sensitivity factor. A Jaszczak phantom was filled with a known activity of $^{177}$Lu before being acquired with the same protocol as for patients [27, 28]. The sensitivity factors in tomographic mode (i.e. taking into account both detector heads) were 7.6 cps/MBq and 18 cps/MBq for the Discovery NM CT 670 and 870DR cameras, respectively. This difference of sensitivity factor can probably be explained by the crystal thickness and energy window. For the early acquisition, approximately one hour after the injection, images from the patient cohort were corrected for dead time according to a variation of the decaying source method. A vial with a known activity of $^{177}$Lu was considered as a point source and then gradually moved away from the detector to vary the count rate as proposed in [29, 30]. For later acquisition, dead time was considered as negligible [31] and not accounted for. Note that the exact time duration between the end of injection and the acquisition was determined according to the acquisition hours stored in the DICOM header of the images and was used for calculations.

### Adaptive organ-based dosimetric workflow

#### General approach

Absorbed dose in organs of interest (kidneys, liver including intra-hepatic lesions, spleen) was estimated from SPECT images as described below. Absorbed dose in bone marrow, which shows a much lower uptake than the other organs, required a specific method that will be described later.

All the four volumes of interest (VOI), left and right kidneys, liver and spleen (LK, RK, L, S), were manually segmented on the first acquired CT image. For liver, if tumors were present, they were included in the segmented volume. Obtained 3D contours were then propagated to all other time-point CT images thanks to deformable vector fields obtained from deformable image registration [32], as described in [33]. Final contours were visually evaluated and manually corrected if necessary.

Monte Carlo simulations with GATE [34] were used to estimate dose rate distributions [33]. To that end, SPECT images were used as a 3D source of emitted gamma. CT images were resampled at the same pixel spacing than the SPECT images (4.4 mm side). SPECT voxels are considered as an isotropic source of decaying $^{177}$Lu with activity proportional to the count value in the voxel. The Monte Carlo engine takes care of simulating all daughter particles, including electrons and photons. The recommended emstandard_opt4 [34] physics list was used. The production cuts were set to 0.1 mm for all particles. Absorbed dose distributions were stored in a 3D matrix of voxels having the same spacing as the initial SPECT image. Simulations were performed to obtain a statistical uncertainty around 1% in each VOI with a high uptake (LK, RK, L, S). In practice, it means that 10$^{6}$ (1 MBq) primary particles (decaying ions) were simulated. The final dose rate distribution was then scaled to the total activity estimated from the SPECT images by summing all voxel values multiplied by the sensitivity factor correspondingly. This computation was performed for all available SPECT time-points (1H, 24H, 96H or 144H). This approach allows us to take into account both self and cross-dose in the MIRD terminology [27].

#### Tri-exponential fit for Time Dose-Rate Curve

**Fig. 1:**
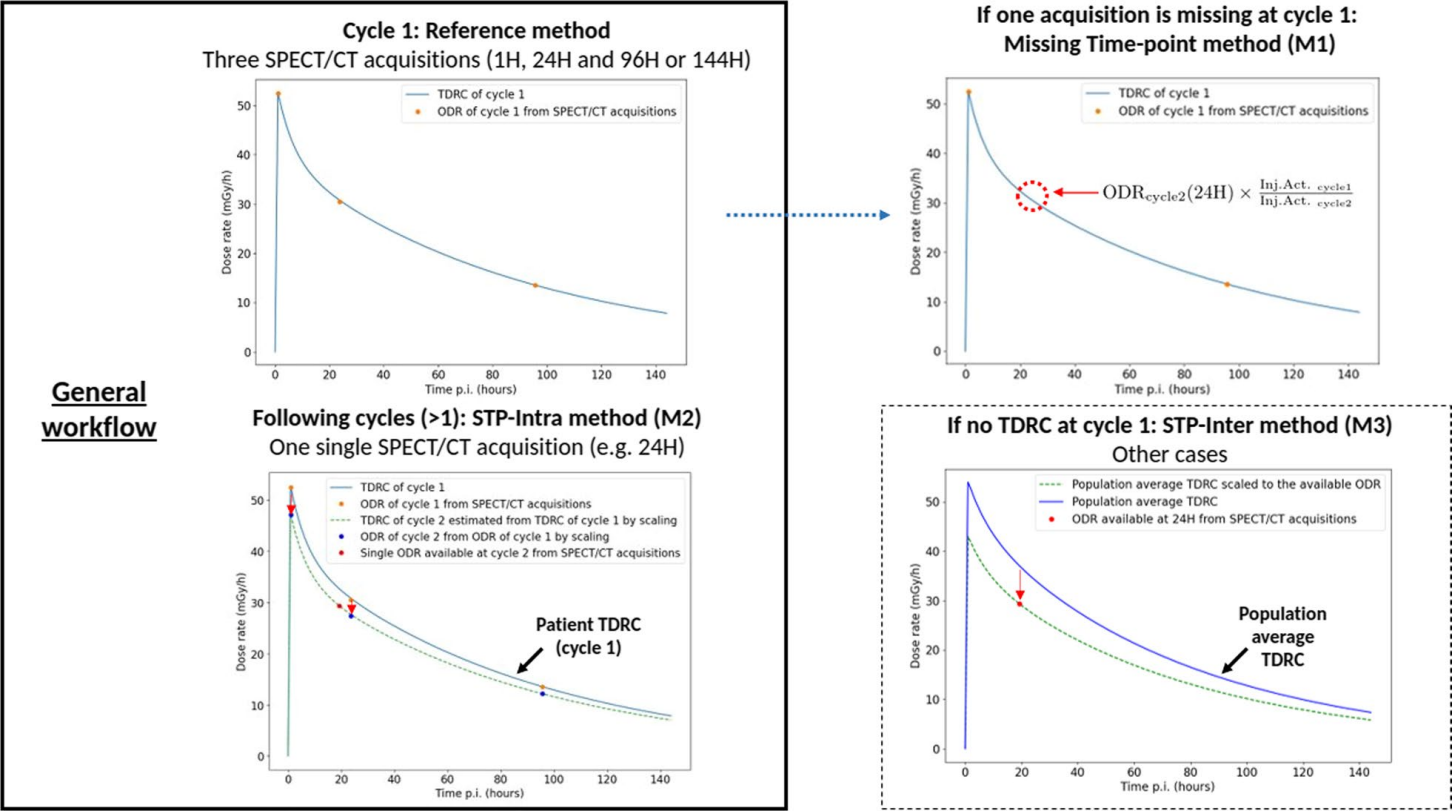
Scheme of the proposed dosimetric workflow illustrated with the left kidney of patient 3 (cycles 1 and 2) of the patient cohort. When three SPECT/CT acquisitions are available at cycle 1, the TDRC was fitted with a tri-exponential function: it is the reference method (at the top left). If one acquisition was missing at cycle 1 but there was an acquisition at the same time-point for a following cycle, the missing ODR was approximated by the ODR of the first next cycle scaled according to injected activities: it is the Missing Time-Point method M1 (at the top right). TDRC of following cycles with one single acquisition was estimated by TDRC at cycle 1 scaled to the single available ODR (all points were scaled by the ratio between the ODR of the single time-point and the ODR computed at the same time on the TDRC): it is the STP-Intra method M2 (at the bottom left). In all other cases, TDRC was estimated by population-averaged TDRC scaled by the latest ODR available: it is the STP-Inter method M3 (at the bottom right)

For all VOI and all time-points, the organ dose rate (ODR) was computed by averaging dose rate values of all voxels belonging to the VOI. Note that dose rate distribution maps were upsampled to the same pixel spacing than VOI drawn on CT images. This step leads to a Time Dose-Rate Curve (TDRC) for all injections, similar to the conventional Time-Activity Curve (TAC) except that it is determined with dose rate and not the activity, thus already integrating the cross-dose. In order to compute the total absorbed dose corresponding to the time integral of those curves, the analytical method proposed by Jackson et al. [35, 36] based on tri-exponential function was used by replacing activity (*A*) with dose rate ($\Omega$), as shown in Eq. (1).

1 $$\begin{aligned} \mathrm{TDRC}(t)\ [\mathrm{Gy/s}] = -\Omega _{1}e^{-(\uplambda +k_{1})t}\ +\ \Omega _{2}e^{-(\uplambda +k_{2})t} +\ \Omega _{3}e^{-(\uplambda +k_{3})t} \end{aligned}$$

where $\uplambda$ is the radioactive decay constant of $^{177}$Lu, $\Omega _{i}$ and $k_{i}$ are the amplitudes and decay rates of three phases: an uptake phase followed by a rapid wash-out and a long-term clearance. An example is available at the top left corner of Fig. 1. Tri-exponential parameters were computed with the same algorithm described in [35]; therefore, all $\Omega _i$ were decay-corrected before fitting TDRC, and the correction was reversed during integration. ODR was assumed to be equal to zero just before the treatment and 600H after the injection. The parameter $k_1$ was set to − 1.3 $s^{-1}$ in the article by Jackson et al. [35]. Here, we are dealing with TDRC and not TAC so this value cannot be used. Instead, the value of $k_1$ was optimized by gradient descend in order to obtain a difference between the ODR value of the first acquisition and the fitted model lower to 0.00001 mGy/s (i.e. 1000 times lower than the ODR value). At the end, the time-integration of TDRC corresponds to the absorbed dose by organ, as shown in Eq. (2).

2 $$\begin{aligned} \mathrm{Dose\, by\, organ\, [Gy]} = \frac{-\Omega _{1}}{(\uplambda \ +\ k_{1})}\ +\ \frac{\Omega _{2}}{(\uplambda \ +\ k_{2})}\ +\ \frac{\Omega _{3}}{(\uplambda \ +\ k_{3})} \end{aligned}$$

#### Particular case: acquisition at 24H was missing in the cycle 1

The tri-exponential function can be used if three ODRs are available as planned in cycle 1. However, the ODR at 24H was missing for some patients. In that case, the missing ODR was substituted with the ODR of another cycle at 24H for the same patient (the closest in time) and its exact time duration was used for the TDRC fitting. Since the injected activities vary from cycle to cycle, the substituted ODR was scaled to the cycle activity. This method assumes that the difference between the missing ODR and the substituted ODR is small; therefore, the closest cycle has been used. This method is named the Missing Time-Point method (M1) and is shown in Fig. 1.

The variability of ODR was assessed for each patient and each VOI at 24H (ex: ODR of left kidney at 24H of cycles 1, 2, 3 and 4). For this, ODR was scaled to the first injected activity, and a variation coefficient (%) was computed.

To estimate the error associated with the Missing Time-Point method (M1), we calculated absorbed doses by organs with two methods for patients who had three ODR: (1) from the three initial ODR (reference method) and (2) from two initial ODR and a substituted ODR at 24h (Missing Time-Point method M1). The percentage of dose difference between both methods was computed for the patient and the validation cohorts.

#### Absorbed dose estimation for cycles with a single acquisition

At cycles 2, 3 and 4, one single ODR at 24H was generally available. Absorbed dose estimation was performed by exploiting tri-exponential-fitted TDRC from the first cycle, assuming similar pharmacokinetics after assessing the variability of the ODR for each time point and each patient. The TDRC was scaled to the single ODR value as illustrated Fig. 1. Time-integrated absorbed dose was computed from the scaled TDRC. This method is named the STP-Intra method for Single Time-Point Intra-patient method (M2). A similar approach was proposed by Garske et al. [21] with a mono-exponential function where they assumed that effective half-life was unchanged between cycles.

For eight patients, three time-points were available for several cycles: one from the patient cohort (cycles 3 and 4) and seven from the validation cohort (cycles 1 and 4). In these cases, the STP-Intra method (M1) was compared to the tri-exponential method (reference method) using three time-points. Percentages of dose difference were computed.

In the rare cases when cycle 1 has not enough time-points, tri-exponential fit cannot be used and absorbed dose was estimated by using the average TDRC of other patients, scaled to the available ODR, like in [22], and is illustrated in Fig. 1. This method assumes that the pharmacokinetics of TDRC are homogeneous from one patient to another. This second alternative method is named the STP-Inter method for Single Time-Point Inter-patient method (M3).

The errors associated with this approach were estimated for cycle 1 in the patient cohort and for cycles 1 and 4 for the validation cohort by leave-one-out, comparing tri-exponential to STP-Inter dose estimation and computing the percentage of dose difference. The leave-one-out method was applied independently to each cohort.

### Dosimetric workflow for bone marrow

For bone marrow, the workflow was similar except that three different VOI types were delineated to estimate doses absorbed for tests, according to various proposals in the literature: (1) the trabecular part of vertebrae between L2 and L4, (2) between L1 and L5 and (3) between T9 and L5. Note that patients in both cohorts did not have bone marrow lesions. In the presence of such lesions, bone marrow substitutes cannot really be used.

As explained in [37], VOI should contain bone marrow (thoracic vertebrae: 16.1% of total bone marrow and lumbar vertebrae: 12.3% in adults) and be in the vicinity of organs with a high uptake because those volumes will mainly absorb the dose (cross-dose). L2 to L4 VOI was used in [38, 39] and was related hematological toxicity. L1 to L5 and T9 to L5 were used by Hagmarker et al. [40] to establish dose/toxicity relationship. For thoracic vertebrae, only vertebrae visible in the field of view of all patients were selected for this study. Dosimetry in all VOI was compared. Moreover, the visible part of the humerus (upper part) was also delineated to have a reference for the background noise because it is the only region where there is no tumor or organ with a high uptake but that contains bone marrow (2.3% [37]). Note that for three patients in the validation cohort, the T9 vertebrae was not or not totally in the field of view.

## Results

### Dosimetric results of the patient cohort

#### Dead time correction

The dead time was 1.87$\upmu$s for the GE Discovery NM CT 670, and a deadtime correction was applied for the patient cohort. The impact of dead time on the absorbed doses appears very limited whatever the volume of interest: less than 3% with a maximum of 6% for bone marrow.

#### Liver, kidneys and spleen

**Fig. 2:**
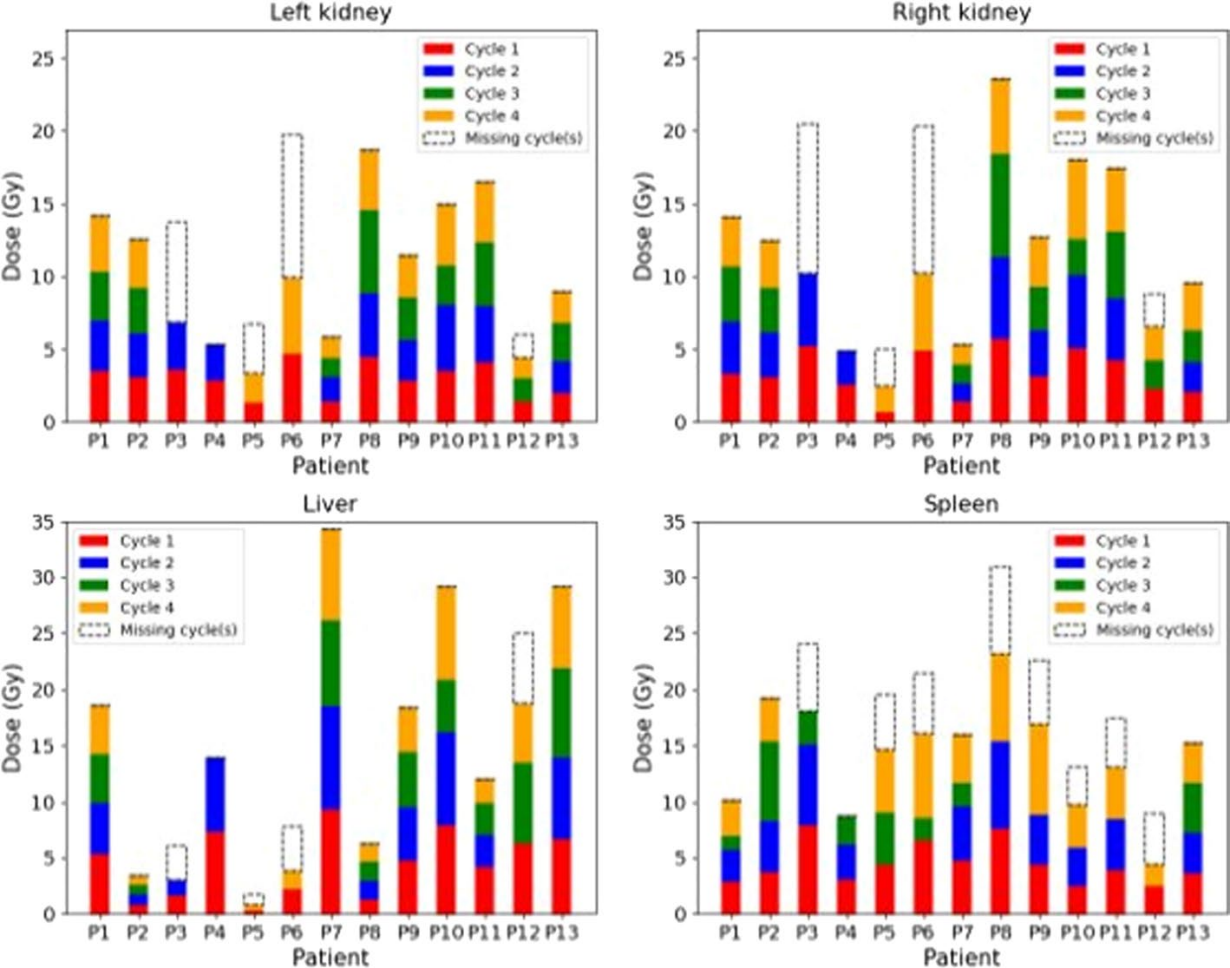
Absorbed doses (Gy) for 13 patients of the patient cohort and for four volumes (left kidney, right kidney, liver and spleen) for all available cycles. For missing cycles, absorbed dose estimations are in white dotted line

Absorbed doses estimated for all patients in the patient cohort for left and right kidneys, liver and spleen are given in Fig. 2. When data were not available (missing acquisitions or cycles due to the COVID pandemic), absorbed doses were approximated by the mean of absorbed doses available multiplied by the number of cycle(s) missing (in white with dotted line). Note that the scale is not the same for each graph.

The *inter-patient* variability of cumulative absorbed doses was large for all organs ([minimum (Gy), median (Gy), maximum (Gy)]: left kidney: [5.9; 13.2; 19.8], right kidney: [5.1; 13.4; 23.6], liver: [1.7; 18.4; 34.3] and spleen: [9.1; 18.6; 31.7]). Overall, the *intra-patient* variability of the absorbed dose for all cycles was low: the mean of variation coefficients of absorbed doses between cycles was: 10.4% for LK, 14.9% for RK, 15.5% for L and 11.5% for S.

**Fig. 3:**
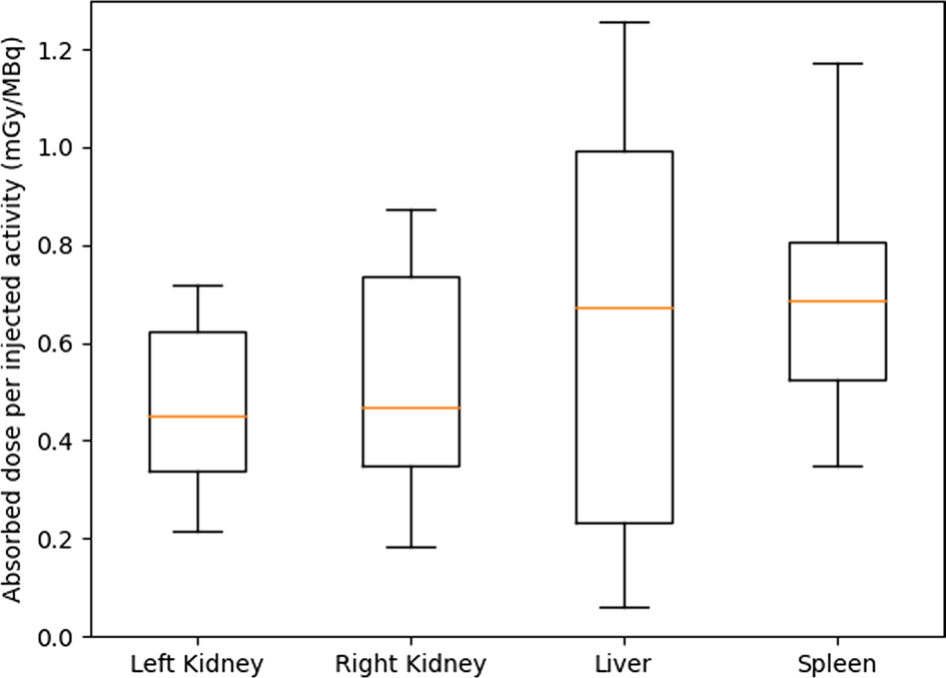
Boxplots representing doses per injected activity (mGy/MBq) averaged on all cycles available for each patient of the patient cohort and each organ (left kidney, right kidney, liver and spleen)

Doses per injected activity [mGy/MBq] were averaged on all cycles for each patient and are represented in Fig. 3 for the four organs. For bone marrow, doses per injected activity were given as mean ± std: 0.04 ± 0.02 mGy/MBq for L2 to L4, 0.04 ± 0.03 mGy/MBq for L1 to L5 and 0.04 ± 0.02 mGy/MBq for T9 to L5.

Coefficients of variation (CV) of doses per injected activity between different cycles were computed for each organ and each patient of the patient cohort. For kidneys and spleen, CV were inferior to 16% in 85% of patients, whereas for liver, L2 to L4, L1 to L5 and T9 to L5, this percentage decreased to 77%, 61%, 69% and 69%, respectively.

**Fig. 4:**
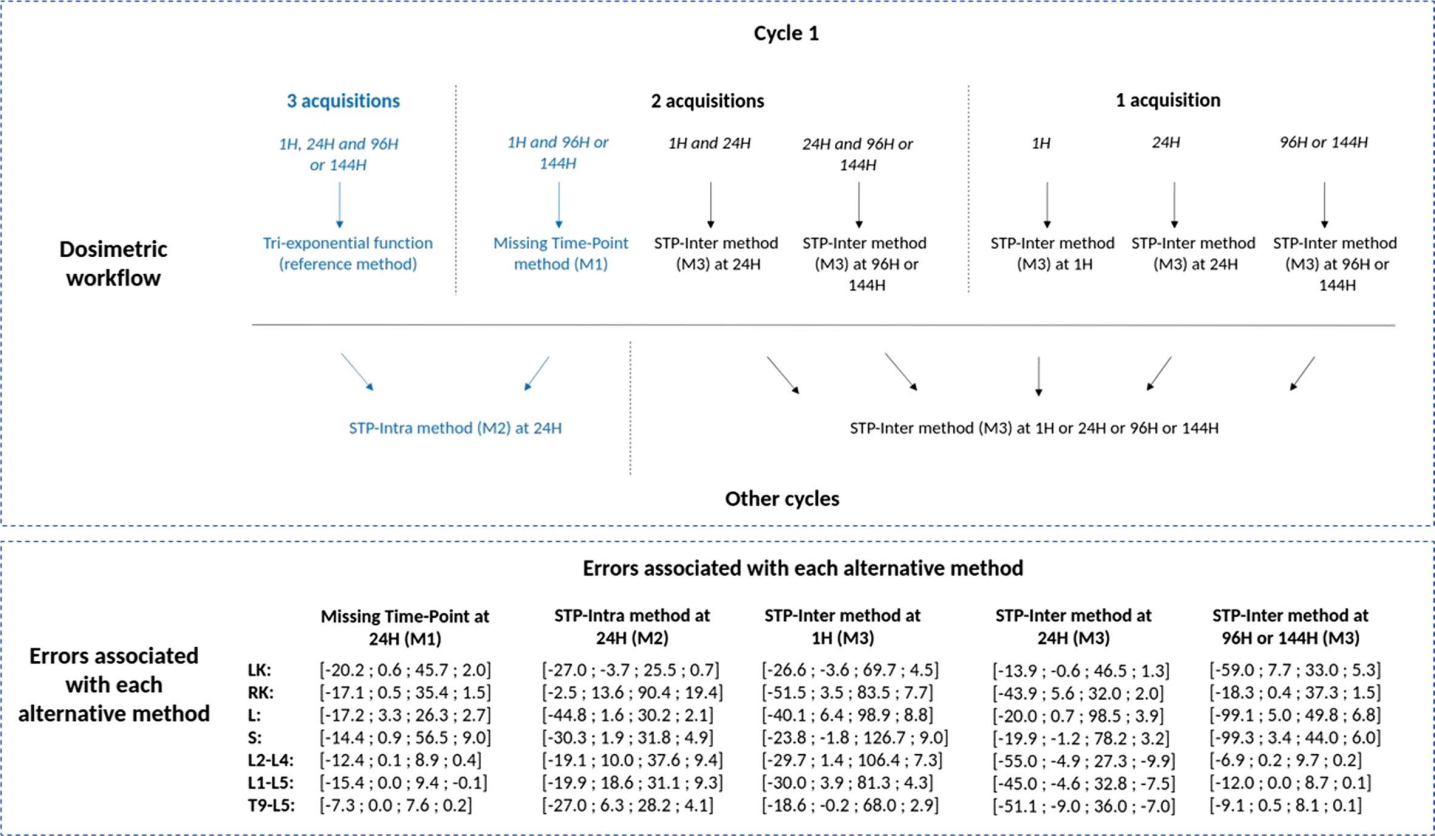
Scheme of the simplified dosimetric workflow used for the estimation of absorbed doses for the patient cohort to the left (LK) and right (RK) kidneys, liver (L), spleen (S) and the three surrogates of bone marrow (L2–L4, L1–L5 and T9–L5). The observed errors were computed on the patient and the validation cohorts and are provided as [minimum; median; maximum; mean]

Finally, Fig. 4 summarizes the workflow used for the patient cohort. The corresponding estimated errors of all cases are proposed below in the form [minimum; median; maximum; mean].

#### Bone marrow

**Fig. 5:**
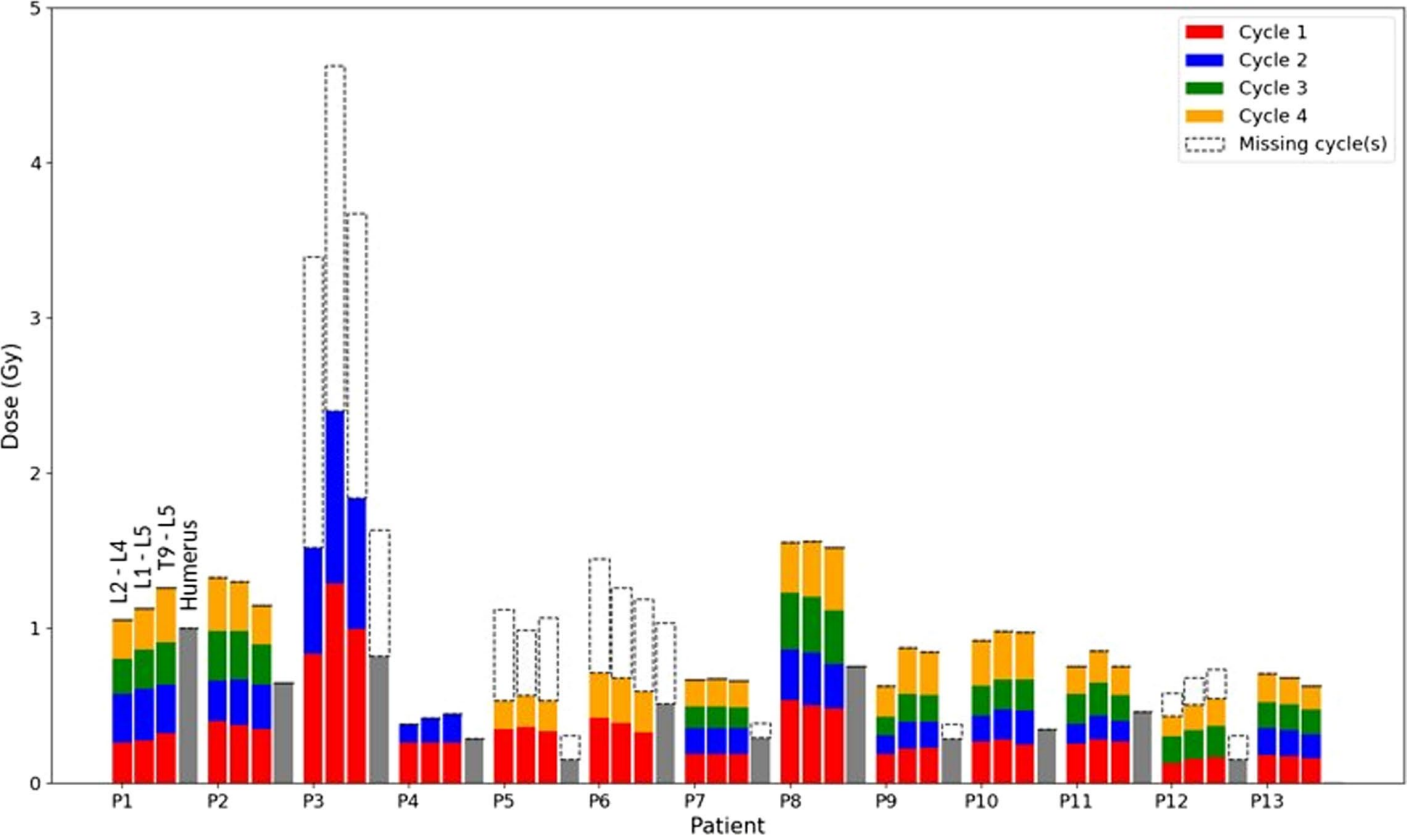
Absorbed doses (Gy) estimated for 13 patients of the patient cohort, for three bone marrow VOI types (trabecular part of vertebrae from L2 to L4, from L1 to L5 and from T9 to L5) and, for humerus as a control. For patient 13, the arms are not in the field of view of any of the images

Absorbed doses estimated for bone marrow are shown in Fig. 5. For each patient, the three first bars represent the absorbed doses measured for each surrogate volume of bone marrow (L2 to L4, L1 to L5 and T9 to L5). The fourth bar depicts the doses absorbed by the humerus as a control. For patient 1, the L5 vertebrae was not visible on the CT image at 96H of cycle 1. The delineation of the humerus depends on the patients and the position of arm on the table and was not possible for few images: for patient 13 for example.

### Comparison of M1, M2 and M3 methods vs reference method (patient and validation cohorts)

#### Variation coefficients of ODR

The variation coefficients of ODR give an indication of differences between ODR at the same time-point but different cycles scaled to injected activities. They were estimated from all patients’ data of the two cohorts at 24H, with the validation cohort and patients 8, 9 and 10 at 1H/6H and with only the validation cohort at 7D. Medians of variations coefficients (%) of ODR are shown in Table 2.

**Table 2:** Medians of variations coefficients (%) of ODR obtained for each VOI and each time-point on all patients (patient and validation cohorts)

Organs	Median (1H/6H) [Min; Max]	Median (24H) [Min; Max]	Median (96H/7D) [Min; Max]
LK	4.0% [0.2; 49.5]	8.2% [1.7; 46.8]	26.1% [1.0; 64.9]
RK	5.0% [1.1; 47.8]	8.0% [1.2; 68.5]	14.3% [0.5; 72.2]
L	15.6% [2.2; 45.7]	9.8% [1.4; 47.9]	19.5% [0.4; 139.9]
S	22.3% [8.4; 49.7]	12.1% [0.3; 58.6]	34.7% [16.4; 140.0]
L2–L4	9.5% [1.1; 35.7]	15.4% [2.8; 43.8]	6.2% [1.2; 43.0]
L1–L5	12.5% [0.7; 31.3]	14.9% [0.5; 36.0]	8.0% [0.5; 40.2]
T9–L5	9.4% [2.4; 33.5]	13.5% [2.8; 34.6]	4.4% [0.4; 15.6]

#### Data used to evaluate simplified methods M1, M2 and M3

To estimate the error associated with each method, only cycles with three SPECT/CT acquisitions were used for the M1 and M3 methods (data of the patients 1, 2, 3, 4, 6, 8 at cycle 1 and patient 10 at cycles 3 and 4 of the patient cohort and all patient’s data of the validation cohort). For the M2 method, two cycles with three SPECT/CT acquisitions were required (data of the patient 10 of the patient cohort and all patient’s data of the validation cohort).

#### Error associated with the Missing Time-Point method (M1) at cycle 1

**Fig. 6:**
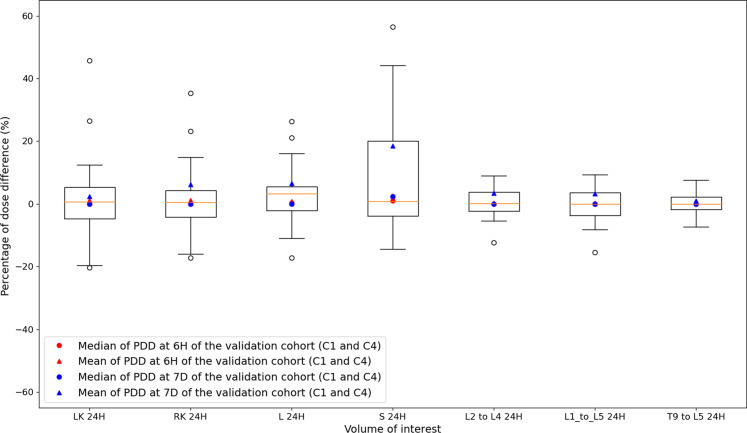
Boxplots of percentages of dose difference (PDD) between absorbed doses estimated with the Missing Time-Point method (M1) at 24H and absorbed doses estimated with the reference method (tri-exponential function for fitting TDRC) for each VOI: LK, RK, L, S, L2 to L4, L1 to L5 and T9 to L5. Each boxplot includes PDD of the patient cohort at 24H (only cycles with three SPECT/CT acquisitions as shown in Table 1) and PDD of the validation cohort at 24H of cycles 1 and 4. For the two other time-points (6H in red and 7D in blue), PDD are estimated from the validation cohort only (cycles 1 and 4), and the mean and the median are represented by triangles and circles, respectively

**Fig. 7:**
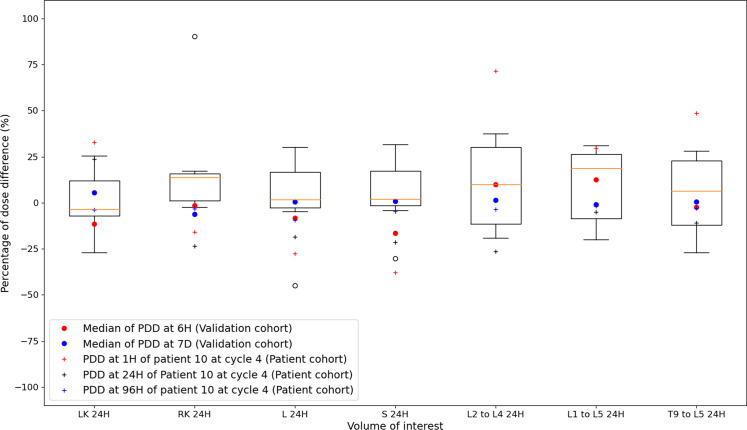
Boxplots of percentages of dose difference (PDD) between absorbed doses estimated with the STP-Intra method (M2) at 24H and absorbed doses estimated with the reference method (tri-exponential function for fitting TDRC) for each VOI: LK, RK, L, S, L2 to L4, L1 to L5 and T9 to L5. Each boxplot includes PDD of the validation cohort at 24H of cycle 4 (TDRC of cycle 1 is used to estimate absorbed doses at cycle 4 from the acquisition at 24H). PDD are also calculated for the patient 10 of the patient cohort between cycles 3 and 4 (red, black and blue crosses for, respectively, 1H, 24H and 96H). For the two other time-points (6H in red and 7D in blue), PDD are estimated from the validation cohort only and medians of PDD are represented by circles

**Fig. 8:**
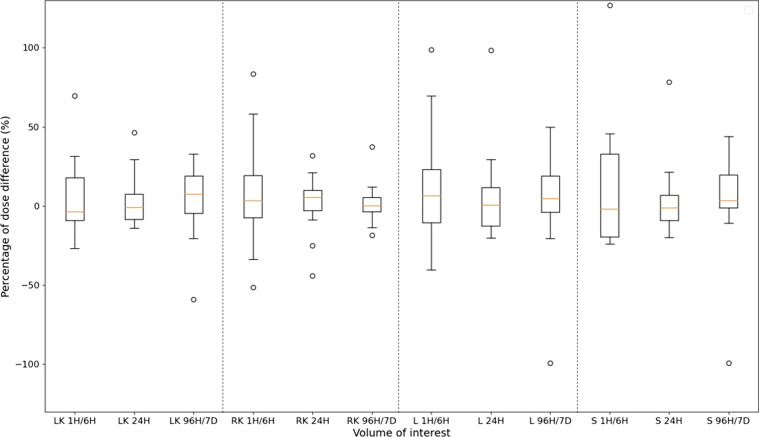
Boxplots of the percentages of dose difference (PDD) between absorbed doses estimated with the STP-Inter method (M3) and with the reference method (tri-exponential function for fitting TDRC) for each time-point (1H/6H, 24H and 96H/7D) and for each VOI with a high uptake (LK, RK, L and S). Each boxplot includes PDD of the patient cohort at each time-point (only cycles with three SPECT/CT acquisitions as shown in Table 1) and PDD of the validation cohort at each time-point of cycles 1 and 4

**Fig. 9:**
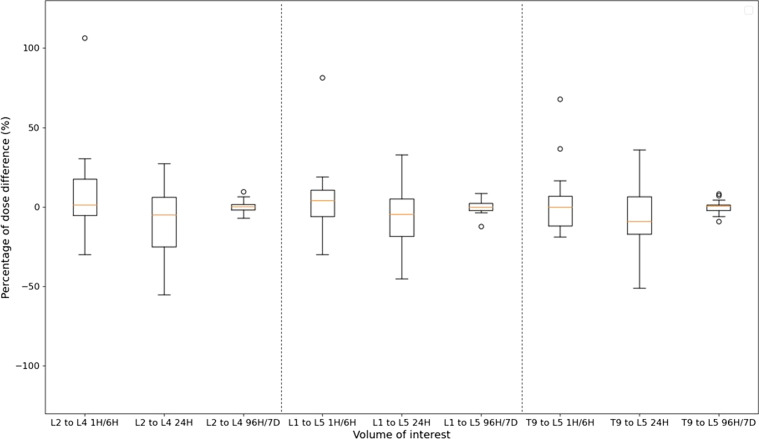
Boxplots of the percentages of dose difference (PDD) between absorbed doses estimated with the STP-Inter method (M3) and with the reference method (tri-exponential function for fitting TDRC) for each time-point (1H/6H, 24H and 96H/7D) and for each surrogate of bone marrow (L2 to L4, L1 to L5, T9 to L5). Each boxplot includes PDD of the patient cohort at each time-point (only cycles with three SPECT/CT acquisitions as shown in Table 1) and PDD of the validation cohort at each time-point of cycles 1 and 4

Figure 6 depicts percentages of dose difference (PDD) between absorbed doses estimated from the Missing Time-Point method (M1) and the reference method (tri-exponential fit) for each VOI and each time-point. Medians were inferior to 1.0%, 3.3% and 2.5% in absolute value when the replaced acquisition was at 1H/6H, at 24H and at 96H/7D whatever the VOI. Standard deviations were between 3.0% and 7.1% at 1H/6H, between 3.8% and 18.9% at 24H and between 5.7% and 31.8% at 96H/7D except for spleen (54.7%) because of the outliers of patient 6 of the validation cohort. Note that the scales are not the same between Fig. 6 and Figs. 7, 8 and 9.

#### Error associated with the STP-Intra method (M2)

Figure 7 depicts PDD between absorbed doses estimated from the STP-Intra method (M2) and the reference method for each VOI and each time-point. The lowest medians are obtained when the single time-point available is at 7 days: they were between − 6.3% and 5.4%, whereas they were between − 16.3% and 12.7% and between − 3.7% and 18.6% for time-points at 6H and 24H, respectively, for the validation cohort only. Medians of surrogates of bone marrow were inferior to 10.0% for L2 to L4, to 18.6% for L1 to L5 and 6.3% for T9 to L5 whatever the time-point. Standard deviations were between 10.4% and 41.9% at 1H/6H, between 17.3% and 32.3% at 24H and between 6.1% and 74.6% at 96H/7D for the validation cohort only. The variability of the PDD of surrogate volumes of bone marrow was almost the same as other VOI at 24H (mean ± std: 0.7 ± 17.3% for LK, 19.4 ± 32.3% for RK, 2.1 ± 25.2% for L, 4.9 ± 20.7% for S, 9.4 ± 23.6% for L2 to L4, 9.3 ± 21.1% for L1 to L5 and 4.1 ± 21.9% for T9 to L5).

#### Error associated with the STP-Inter method (M3)

Figures 8 and 9 represent PDD between absorbed doses estimated with the STP-Inter method (M3) and the reference method using leave-one-out cross-validation, for the three time-points 1H/6H, 24H and last one (96H or 144H or 7D), and for all VOI (LK, RK, L and S in Fig. 8 and L2–L4, L1–L5 and T9–L5 in Fig. 9). Medians (standard deviation) were between − 3.6% and 7.7% (14.9% and 21.6%) for LK, 0.4% and 5.6% (11.3% and 29.0%) for RK, 0.7% and 6.4% (25.9% and 33.0%) for L, − 1.8% and 3.4% (20.9% and 36.9%) for S, − 4.9% and 1.4% (4.2% and 27.4%) for L2 to L4, − 4.6% and 3.9% (4.1% and 21.1%) for L1 to L5 and − 9.0% and 0.5% (4.0% and 20%) for T9 to L5 regardless of the time. The variability seems to be lowest for surrogates of bone marrow for late time-points: for example, for L2 to L4, mean and standard deviation of PDD were equal to 1.4 ± 27.4% at 1H/6H, − 4.9 ± 23.3% at 24H and 0.2 ± 4.2% at 96H/7D.

## Discussion

In this work, we proposed an image-based patient-specific dosimetric workflow adaptable to a limited number of SPECT time-points, e.g., three for the first cycle and a single one for the other cycles. It is based on tri-exponential fitting of Monte Carlo computed dose-rate images. It can be used in a clinical context and allows to perform dosimetry even if some acquisitions are missing.

*Dosimetric workflow* Several authors proposed methods to estimate absorbed doses from a low number of acquisitions [15–17, 19–22] by using an approximation of time-integrated activity from mono-exponential model [16, 17] or a bi-exponential model [20] or by exploiting a priori information about pharmacokinetic properties (e.g., pharmacokinetic parameters are homogeneous between patients). In this study, the tri-exponential model was chosen to take into account patients’ physiology and seems to be better than the mono-exponential model [41]. Delker et al. [42] proposed to not use early acquisitions in order to reduce the dose estimation error. In our case, the comparison of the estimated doses with a tri-exponential compared to mono-exponential function with acquisitions at 24H and 96H/144H shows that the dose difference is small (< 3%) for kidney, liver and spleen but can be as high as 3000% if the ODR at 24H is less than or equal to the ODR at 96H/144H for bone marrow substitutes (low activity in these regions). An early acquisition is necessary to estimate the uptake phase, which is very fast. During the 1H imaging session, the distribution in the patient may change rapidly compared to the acquisition time. However, the rotating acquisition of 15 min (one bed) tends to smooth out the activity, and we did not observe large distribution changes between the first and the last projections of the sequence.

**Fig. 10:**
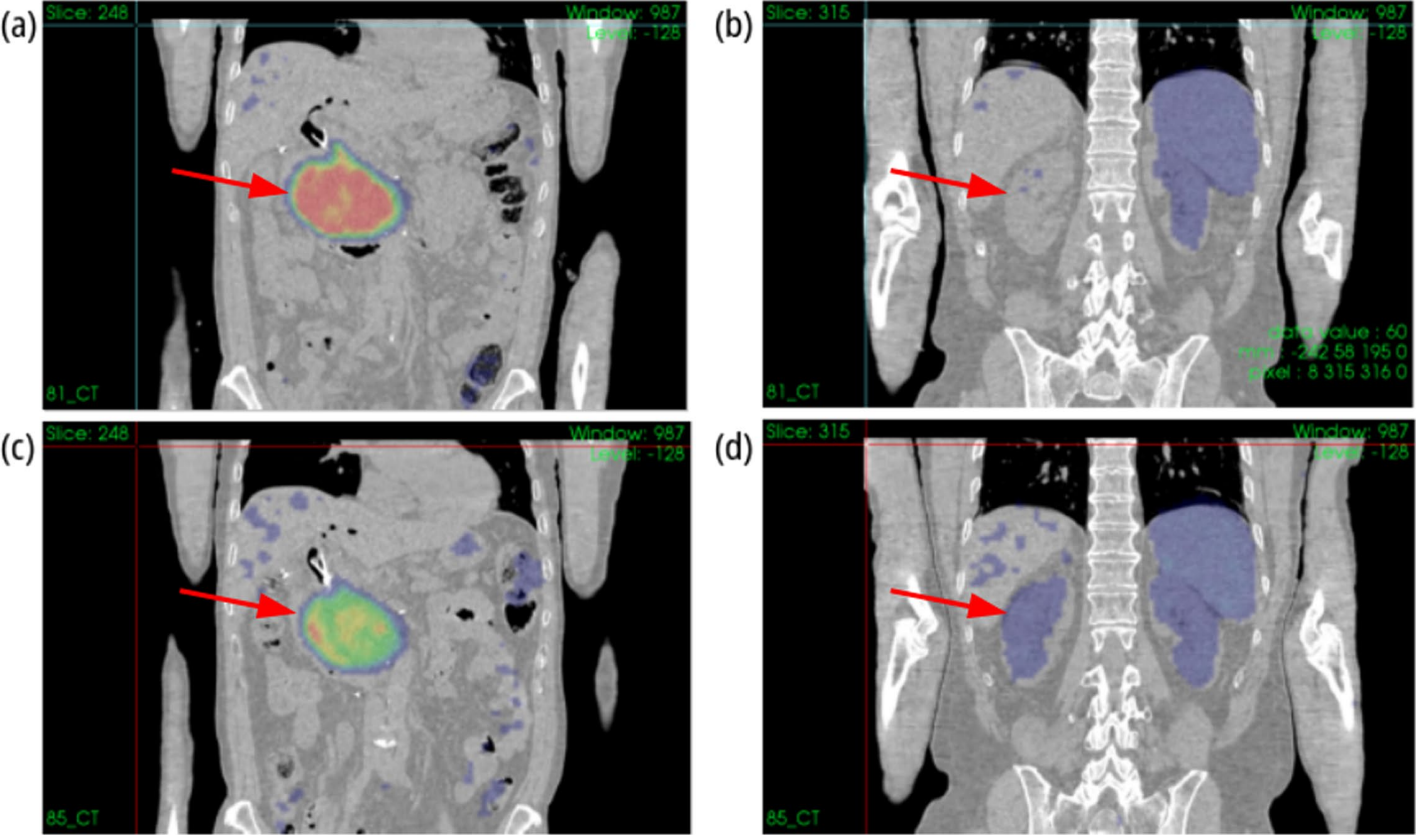
SPECT images superimposed on CT scans for patient 5 of the patient cohort at cycles 1 (**a**, **b**) and 4 (**c**, **d**) showing the evolution of biodistribution of activity from one cycle to another at the lesion level (**a**, **c**) and at the right kidney level (**b**, **d**). The same color scale was used for all images

Moreover, dosimetry was performed with Monte Carlo simulations to take into account patients’ images and cross-irradiation [43, 44]. The cross-dose contribution is significant for bone marrow [45] and may be significant for kidneys if its self-absorbed dose is low or if the tumor burden is high [46]. It is the case of patient 5 presented in Fig. 10 who has a large isolated tumor that contributes more than 93% to the bone marrow dose, 2% and 50% to the left and right kidneys. The computational time was larger than analytical approaches (local deposition method or DPK convolution), approximately 10 minutes of computation time on a conventional workstation for each acquisition. For low activity regions (such as vertebrae), 1 hour computation time (5 $\times$ 10$^{6}$ simulated primary decays on a single core) is necessary to reach less than 5% uncertainty in all VOI.

*Dosimetry* Inter-patient variability in the doses absorbed by each organ (Fig. 2) was high: coefficients of variations of cumulative absorbed doses were 38.4% for LK, 43.6% for RK, 70.9% for L and 38.3% for S (when patient 4 was excluded, two cycles only). This inter-patient variability can be partly explained by the proximity of lesions or organs with a high uptake (liver for the right kidney and spleen for the left kidney). For example, doses to the liver were 3.4 Gy for P2 and 34.3 Gy for P7 (all four cycles were taken into account) because the latter depicted intra-hepatic lesions. This variability illustrates the importance of performing personalized dosimetry to analyze treatment effects.

The intra-patient inter-cycle variability is illustrated in Figs. 2 and 5. It was relatively low for organs with high uptake: coefficients of variation of doses per injected activity were inferior to 16% for 85% of patients for LK, RK and S and 77% of patients for L. However, this was not the case for bone marrow (61% for L2 to L4, 69% for L1 to L5 and for T9 to L5). Sundlöv et al. [47] calculated biologically effective dose and also observed large inter-cycle variability in the kidneys for different patients. They supposed that these changes came from the blood pressure and degree of hydration [47]. In their study, medians of absorbed doses per activity injected were, for LK and RK, close to the ones found here (medians were 0.45 mGy/MBq and 0.47 mGy/MBq for LK and RK, respectively). For patient 5, CV were higher for all VOI: between 17.6% and 68.5%. This variation is related to observed changes in the activity distribution between cycles 1 and 4: indeed, activity decreases in a large lesion and increases in the right kidney and the liver as shown in Fig. 10 (in Supp. Materials).

For OAR, maximum tolerated dose (MTD) thresholds from EBRT have been considered: 23 Gy for kidneys and 2 Gy for bone marrow [4]. For kidneys, one patient (P8 with 19 Gy and 24 Gy for LK and RK, respectively) exceeded the threshold if we consider missing cycles too, but no severe renal toxicity was observed. Sundlöv et al. [47] chose different thresholds for patients with and without risk factors (27 Gy and 40 Gy, biologically effective dose), and they observed a moderate decline of renal function after treatment, some patients receiving up to 8 cycles of 7.4 GBq.

In order to estimate the absorbed doses by bone marrow, three VOI were delineated as surrogates instead of using blood samples or biopsy [48]. The normality of the series of cumulative absorbed doses (only available cycles) was assessed with a Shapiro–Wilk test (*p* value = 0.1038 for L2 to L4, *p* value = 0.01875 for L1 to L5 and *p* value = 0.07838 for T9 to L5). The difference observed between the series of accumulated absorbed doses of L2 to L4 and L1 to L5 was significant (Wilcoxon signed-rank test: *p* value = 0.02), which was not the case for the other comparisons (Wilcoxon signed-rank test: *p* value = 0.45 between L2 to L4 and T9 to L5 and *p* value = 0.09 between L1 to L5 and T9 to L5). When comparing the three types of VOI, the cumulative absorbed doses were close (maximum difference: 0.2 Gy in absolute value) except for the patient 3 (maximum difference: 0.9 Gy in absolute value). In this study, patients did not have bone marrow lesions, but for patients who did, the dosimetry with BM substitutes would need to be changed. This is, for example, the case of patients treated with $^{177}$Lu-PSMA. For patient 3, the absorbed doses have been evaluated over two cycles only and the 2 Gy limit was already almost reached. It is likely that the limit was reached with the two other cycles, but no significant toxicity was observed. In contrast, patient 10 shows grade 2 toxicity for platelets and for leukocytes and grade 3 toxicity for lymphocytes after cycle 2, while MTD was not reached, which led to a halving of the injected activity.

Finally, doses in bone marrow surrogates were compared to the dose absorbed by the upper part of the humerus to assess that the dose estimates in the vertebrae were different than the activity in the blood. Cumulative absorbed doses (only cycles where humerus is visible) were significantly different for the three surrogates of bone marrow (Wilcoxon signed-rank test: *p* value = 0.0005 for L2 to L4, *p* value = 0.0005 for L1 to L5 and *p* value = 0.0005 for T9 to L5).

*Evaluation of the uncertainties* The proposed methods are based on tri-exponential fit and assumed that pharmacokinetics remains approximately the same from one cycle to another for the same patient. Calculations of variation coefficients were performed in the worst case (ODR of cycles 1 and 4 only for the validation cohort), and the medians of variation coefficients were limited at 15.4% at 24H (22.3% at 1H/6H and 34.7% at 96H/7D). The assumption seems to be globally verified, until there is no significant toxicity or a significant variation in the distribution of activity. For P5 of the patient cohort, the activity in the lesion decreased between cycles 1 and 4 in contrast to the activity in right kidney and liver: CV were, respectively, 68.5% and 47.9%. Overall, the impact on the percentage of dose difference (PDD) remains moderate: medians of PDD were inferior to 3.3% whatever the VOI and the time-point. As a conclusion, replacing the 24H dose-rate with the one of another cycle allowing tri-exponential fit seems a reasonable workaround. For cycles with one acquisition, two methods were proposed: STP-Intra (M2) method and STP-Inter method (M3). The lowest median errors were estimated when the acquisition takes place at 96H, 144H or 7D whatever the VOI with the STP-Intra method (M2). With the STP-Inter method (M3), it does not seem to be a preferred time-point unless one wishes to limit the error made in dose estimation at the bone marrow level in which case a late time-point should also be selected. This observation has also been made by other authors such as [49]. However, delayed acquisitions (96H or 144H) may not always be possible, for example when patients live far from the hospital or due to logistical issues. For the STP-Intra method (M2) only, the errors made with 24H acquisition are smaller than those made at 1H for kidney, liver, spleen and bone marrow. The STP-Intra method (M2) was preferred to the STP-Inter method (M3) because if the patient’s TDRC is not homogeneous with the TDRC of other patients, then the error is likely to be large, as explained in [11, 22]. For example, for the P7 of the validation cohort, PDD of the liver for cycle 4 were equal to − 9.8%, − 1.0% and 1.1% for 6H, 24H and 7D with the STP-Intra method (M2) and − 40.0%, − 20.0% and 15.5% with the STP-Inter method (M3).

The proposed methods were evaluated for all VOI, including bone marrow, while other studies are mostly focused on kidneys only [15, 16, 19, 20] or do not include bone marrow [17, 21, 22]. For bone marrow, three surrogates VOI were assessed. The errors appear to be lower for T9 to L5 with the STP-Intra method (M2), whereas they are equivalent whatever the VOI with the STP-Inter method (M3). The variability of absorbed doses in bone marrow is higher with the STP-Intra method (M2) than with the STP-Inter method (M3) as illustrated in Figs. 7, 8 and 9. One hypothesis could be that this variability is linked to the volume delineation. Indeed, ODR corresponds to the mean of dose rates in the VOI. If the delineation included voxels that are not actually in the volume and which have lower dose rates than those in the VOI, the ODR will be impacted.

*Limits and recommendations* This workflow was assessed for twenty patients (thirteen in the patient cohort and seven in the validation cohort) treated with $^{177}$Lu-DOTATATE. At cycle 1, TDRC were modeled with a tri-exponential function, which was considered as reference. For other cycles, they were estimated from the TDRC of the first cycle by assuming a conservation of pharmacokinetics parameters. However, the preservation of the patient’s physiology may not really be verified if an important toxicity occurs or if the distribution of activity in the patient changes significantly between cycles. It may therefore be interesting to add acquisitions in the event that toxicities occur. Errors committed with each method (Missing Time-Point M1, STP-Intra M2 and STP-Inter M3) depend on the chosen time-point: we recommend to select a late time-point in order to limit the error made in particular with the STP-Intra method (M2). However, in clinical practice, these time-points can be the most difficult to obtain because they require the patient to return to the hospital: that’s why we continue to acquire a single SPECT/CT image at 24H despite the results. The STP-Inter method is an alternative method when the standard method (tri-exponential fitting or STP-Intra method M2) cannot be applied, but it has two major disadvantages: it requires the TDRC of other patients to be available prior to dosimetry and errors committed are linked to the homogeneity of the patient and cohort TDRC. The STP-Intra method should be preferred as much as possible in order to limit the errors made.

## Conclusion

This study proposes a patient-specific image-based dosimetric workflow applicable in clinic, based on adaptive tri-exponential fitting process of Monte Carlo computed dose-rate distributions at organ level. It can be used for any VOI, including bone marrow, and has been applied to patients treated with $^{177}$Lu-DOTATATE. The workflow adapts to a different number of SPECT/CT acquisitions per cycle in particular when acquisitions are missing, preserves the patient’s physiology and automatically accounts for cross-irradiation caused by lesions of different shape, fixation and location. All methods are associated with an estimate of the error made.

## Data Availability

GATE scripts for simulation during the current study are available from the corresponding author on reasonable request.
